# Sex and age differences in isolated traumatic brain injury: a retrospective observational study

**DOI:** 10.1186/s12883-021-02305-6

**Published:** 2021-07-05

**Authors:** Sanae Hosomi, Tetsuhisa Kitamura, Tomotaka Sobue, Hiroshi Ogura, Takeshi Shimazu

**Affiliations:** 1 Department of Traumatology and Acute Critical Medicine, Osaka University Graduate School of Medicine, 215, Yamada-oka, Suita, Japan; 2 Division of Environmental Medicine and Population Sciences, Department of Social and Environmental Medicine, Osaka University Graduate School of Medicine, 215, Yamada-oka, Suita, Japan

**Keywords:** Mortality, Traumatic brain injury, Sex, Epidemiology, In-hospital complications

## Abstract

**Background:**

Among the many factors that may influence traumatic brain injury (TBI) progression, sex is one of the most controversial. The objective of this study was to investigate sex differences in TBI-associated morbidity and mortality using data from the largest trauma registry in Japan.

**Methods:**

This retrospective, population-based observational study included patients with isolated TBI, who were registered in a nationwide database between 2004 and 2018. We excluded patients with extracranial injury (Abbreviated Injury Scale score ≥ 3) and removed potential confounding factors, such as non-neurological causes of mortality. Patients were stratified by age and mortality and post-injury complications were compared between males and females.

**Results:**

A total of 51,726 patients with isolated TBI were included (16,901 females and 34,825 males). Mortality across all ages was documented in 12.01% (2030/16901) and 12.76% (4445/34825) of males and females, respectively. The adjusted odds ratio (OR) of TBI mortality for males compared to females was 1.32 (95% confidence interval [CI], 1.22–1.42]. Males aged 10–19 years and ≥ 60 years had a significantly higher mortality than females in the same age groups (10–19 years: adjusted OR, 1.97 [95% CI, 1.08–3.61]; 60–69 years: adjusted OR, 1.24 [95% CI, 1.02–1.50]; 70–79 years: adjusted OR, 1.20 [95% CI, 1.03–1.40]; 80–89 years: adjusted OR, 1.50 [95% CI, 1.31–1.73], and 90–99 years: adjusted OR, 1.72 [95% CI, 1.28–2.32]). In terms of the incidence of post-TBI neurologic and non-neurologic complications, the crude ORs were 1.29 (95% CI, 1.19–1.39) and 1.14 (95% CI, 1.07–1.22), respectively, for males versus females. This difference was especially evident among elderly patients (neurologic complications: OR, 1.27 [95% CI, 1.14–1.41]; non-neurologic complications: OR, 1.29 [95% CI, 1.19–1.39]).

**Conclusions:**

In a nationwide sample of patients with TBI in Japan, males had a higher mortality than females. This disparity was particularly evident among younger and older generations. Furthermore, elderly males experienced more TBI complications than females of the same age.

**Supplementary Information:**

The online version contains supplementary material available at 10.1186/s12883-021-02305-6.

## Background

Traumatic brain injury (TBI) is a major cause of mortality and morbidity worldwide [1]. While it predominantly affects younger individuals, its incidence is increasing among the 65 years and older age group, especially in developed countries [2].

While prior studies have reported a sex difference in TBI mortality, a recent review of clinical and epidemiological studies concluded that there was insufficient evidence to confirm such a disparity [3]. The results of epidemiological studies on TBI have often been limited in terms of their generalizability to larger populations, due to the large heterogeneity in their patient samples; this has been a source of conflicting results across studies. Certain factors may increase the risk of TBI, owing to their interactions with biological, behavioral, social, and cultural conditions before and at the time of injury [4]. For example, in developed countries, the greater risk of TBI in individuals over 65 years of age has been attributed to a high incidence of falls from low heights. In contrast, decreases in high-velocity traffic accidents, improved road conditions, improved safety features in newer vehicles, and the stronger enforcement of traffic regulations have been reported to be responsible for a lower risk of TBI in the 15- to 44-year-old age group [5]. Therefore, due to an aging demographic, there is an urgent need to fully elucidate age and sex disparities in TBI incidence and outcomes.

The primary objective of this study was to assess sex differences in mortality, following age stratification, among patients treated for TBI in trauma centers. The secondary objective was to characterize sex differences in TBI complications. While the assessment of outcomes following TBI should ideally take into account the features of the specific injury (e.g., mechanism and type of injury, post-injury complications, extracerebral trauma accompanying TBI), we examined isolated TBI in order to eliminate the potential confounding effect of the severity of other associated injuries.

## Methods

### Study design, population, and setting

This nationwide retrospective cohort study was conducted using the Japanese Trauma Data Bank (JTDB). We included patients with TBI who were registered in the database between January 2004 and December 2018, and transported to a JTDB-associated hospital for treatment. TBI was defined as an injury to the brain due to an external force. Patients with TBI were screened with the Abbreviated Injury Scale (AIS) code [6]; we excluded cases with AIS ≥ 3 for all other body regions (chest, abdomen, and extremity), as polytrauma associated with TBI is known to increase the risk of mortality [7]. Furthermore, we excluded patients with a maximum head AIS score of 6 (lethal injury) or 9 (unspecified injury); cardiopulmonary arrest on hospital arrival; a requirement for inter-hospital transport [8, 9]; or missing data for variables required for the logistic regression analysis. Cardiopulmonary arrest was defined as a systolic blood pressure of 0 mmHg and/or heart rate of 0 bpm [10].

### The data registry

The JTDB was launched in 2003 by the Japanese Association for the Surgery of Trauma (Trauma Surgery Committee) and the Japanese Association for Acute Medicine (Committee for Clinical Care Evaluation) [11], and is similar to the trauma databases in North America, Europe, and Oceania. By 2018, a total of 272 major emergency medical institutions across Japan were registered in the JTDB database [10]. The included hospitals have service levels similar to those of level I trauma centers in the United States. Data are collected from participating institutions via the internet, and physicians and medical assistants who attend the AIS coding course are the main contributors of the inputted data [10, 11]. Patient data recorded in the JTDB includes the following: age, sex, mechanism of injury, AIS code (1998 version), injury severity score, vital signs on hospital arrival, date and time series from hospital arrival to discharge, medical procedures (e.g., interventional radiology, surgery, computed tomography), complications, and the date of mortality or hospital discharge [10, 11]. This study used the most recent data available in the JTDB registry.

### Study endpoints

The primary outcome was death at hospital discharge. The secondary outcomes were post-TBI in-hospital neurologic and non-neurologic complications that were diagnosed by an in-hospital medical team.

### Statistical analysis

Patient characteristics were compared between groups using the unpaired t-test for continuous variables and the chi-square test or Fisher’s exact test for categorical variables. A multiple logistic regression analysis was used to assess factors potentially associated with mortality; results were expressed as odds ratios (ORs) and 95% confidence intervals (CIs). Potential confounders included factors that were clinically essential and previously reported to be associated with variables in the multivariable analyses [6, 12, 13]. The multivariable logistic regression model for mortality due to isolated TBI was adjusted for the following variables: age (10-year strata); year of TBI onset (2004–2006, 2007–2009, 2010–2012, 2013–2015, 2016–2018); mechanism of trauma (motor vehicle driver, motor vehicle passenger, back seat passenger, motorcycle driver, motorcycle passenger, bicycle, pedestrian, other vehicle, fall from a great height, fall down the stairs, fall on the ground level, other blunt injury, penetrating); alcohol drinking (no/yes); use of anticoagulant or antiplatelet drugs (no, yes); hypotension on admission to the emergency department (no, yes); Glasgow Coma Scale [GCS] group on arrival [3–15]; maximum head AIS [3–5]; type of TBI (diffuse brain injury, focal brain injury, uncategorized); site of TBI (1, 2, ≥ 3); whether an operation was indicated for TBI (no, yes), and post-TBI complications (none, neurological, non-neurological). In addition, subgroup analyses were performed to identify mortality tendencies at hospital discharge in the different age groups. The OR for mortality in males versus females was determined for each 10-year age stratum. As per the secondary outcome, we used a univariable analysis to compare post-TBI complications in each organ system between females and males in predetermined age groups (0–19, 20–59, ≥ 60). Detailed comparisons of the mechanism and type of TBI by age group were also performed.

Statistical significance was defined as a two-sided p-value of less than 0.05, or assessed using a 95% CI. All analyses were performed using STATA version 16 (StataCorp). This study was reported in accordance with the Strengthening the Reporting of Observational Studies in Epidemiology statement [14].

## Results

**Fig. 1:**
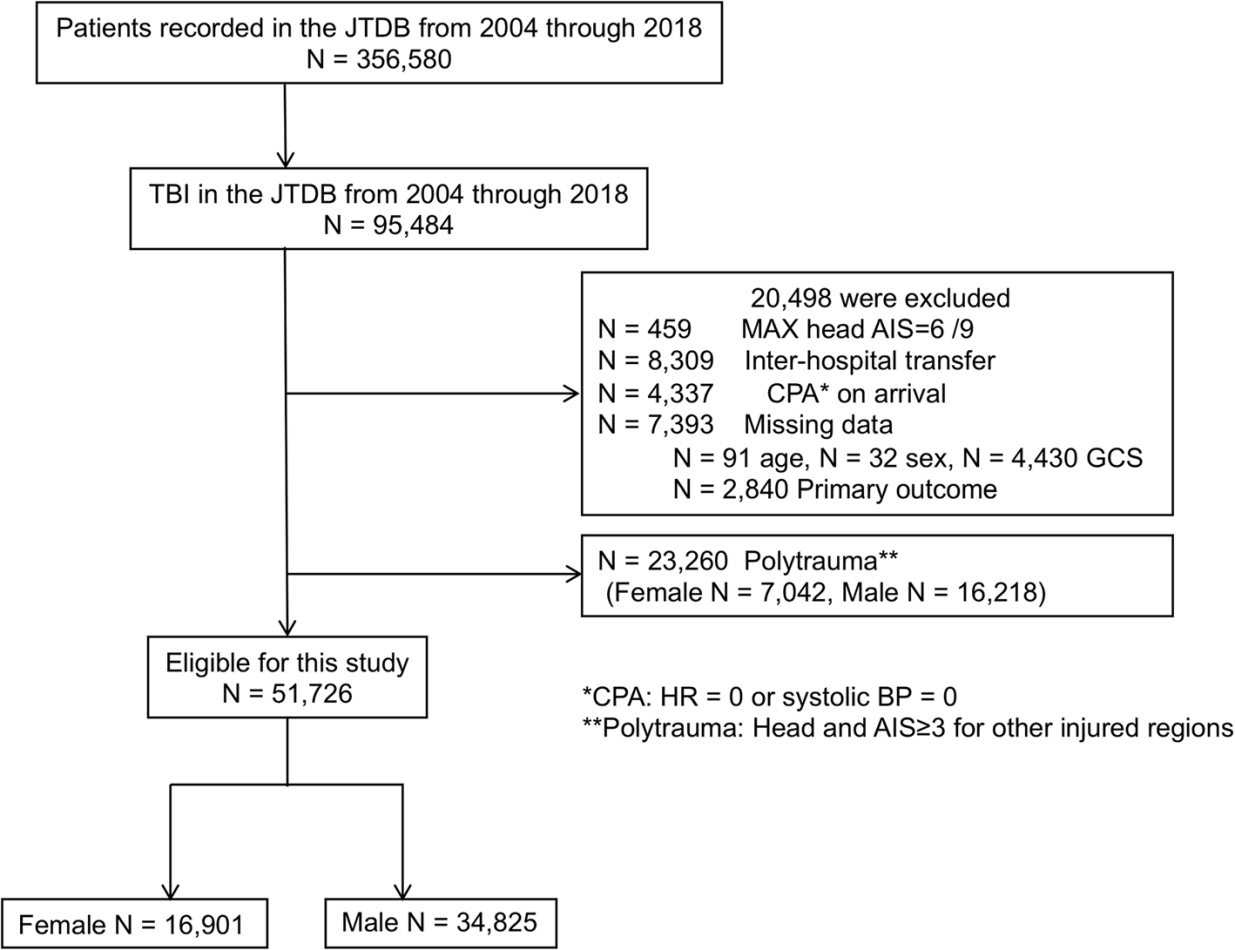
Flow of patients in the study. Of the 51,726 patients included for isolated TBI, there were 34,825 (67.3%) men and 16,901 (32.7%) women. Of the 23,260 patients excluded for polytrauma, there were 16,218 (69.7%) men and 7,042 (30.3%) women. JTDB, Japan Trauma Data Bank; AIS, Abbreviated Injury Scale; CPA, cardiopulmonary arrest; GCS, Glasgow Coma Scale; MAX, maximum; TBI, traumatic brain injury

A total of 51,726 patients with isolated TBI were included in the analysis. Among these patients, 16,901 (32.67%) were females and 34,825 (67.33%) were males. Figure 1 and Additional file 1 depict the flow of patients in the study and the distribution of isolated TBI cases across all ages, respectively. Patient characteristics are summarized in Table 1. The median (interquartile range) age was higher in females (72 (54–82) years) compared to males (64 (42–77) years) (Table 1).

**Table 1:** Comparison of baseline characteristics between males and females with isolated TBI

		Total	Female	Male	*p*-value
		*N* = 51,726	*N* = 16,901	*N* = 34,825	
**Age, year**	median (IQR)	67 (45–79)	72 (54–82)	64 (42–77)	< 0.001
**Year of onset**					
2004–2006	n (%)	1,904 ( 3.7%)	613 ( 3.6%)	1,291 ( 3.7%)	0.004
2007–2009	n (%)	5,990 (11.6%)	1,855 (11.0%)	4,135 (11.9%)	
2010–2012	n (%)	11,576 (22.4%)	3,743 (22.1%)	7,833 (22.5%)	
2013–2015	n (%)	17,045 (33.0%)	5,571 (33.0%)	11,474 (32.9%)	
2016–2018	n (%)	15,211 (29.4%)	5,119 (30.3%)	10,092 (29.0%)	
**Cause of trauma**					
Motor vehicle driver	n (%)	1,822 (3.5%)	445 (2.6%)	1,377 (4.0%)	< 0.001
Motor vehicle passenger	n (%)	313 ( 0.6%)	156 ( 0.9%)	157 ( 0.5%)	
Back seat passenger	n (%)	455 ( 0.9%)	241 ( 1.4%)	214 ( 0.6%)	
Motorcycle driver	n (%)	3,834 ( 7.4%)	793 ( 4.7%)	3,041 ( 8.7%)	
Motorcycle passenger	n (%)	194 ( 0.4%)	80 ( 0.5%)	114 ( 0.3%)	
Bicycle	n (%)	6,539 (12.6%)	2,650 (15.7%)	3,889 (11.2%)	
Pedestrian	n (%)	5,194 (10.0%)	2,365 (14.0%)	2,829 ( 8.1%)	
Other vehicle	n (%)	225 ( 0.4%)	49 ( 0.3%)	176 ( 0.5%)	
Fall from a great height	n (%)	3,067 ( 5.9%)	497 ( 2.9%)	2,570 ( 7.4%)	
Fall down the stairs	n (%)	8,621 (16.7%)	2,451 (14.5%)	6,170 (17.7%)	
Fall on the ground level	n (%)	16,323 (31.6%)	5,926 (35.1%)	10,397 (29.9%)	
Other blunt injury	n (%)	5,086 ( 9.8%)	1,235 ( 7.3%)	3,851 (11.1%)	
Penetrating	n (%)	53 ( 0.1%)	13 ( 0.1%)	40 ( 0.1%)	
**Alcohol drunk**	n (%)	7,957 (15.4%)	979 ( 5.8%)	6,978 (20.0%)	< 0.001
**Anticoagulant / antiplatelet**	n (%)	1,509 ( 2.9%)	495 ( 2.9%)	1,014 ( 2.9%)	0.91
**Hypotension (BP ≤ 90) on arrival**	n (%)	1,395 ( 2.7%)	495 ( 2.9%)	900 ( 2.6%)	0.023
**GCS score on arrival**	median (IQR)	14 (9–15)	14 (10–15)	14 (9–15)	< 0.001
Severe (3–8)	n (%)	11,466 (22.2%)	3,474 (20.6%)	7,992 (22.9%)	< 0.001
Moderate (9–12)	n (%)	6,989 (13.5%)	2,078 (12.3%)	4,911 (14.1%)	
Mild (13–15)	n (%)	33,271 (64.3%)	11,349 (67.1%)	21,922 (62.9%)	
**Type of TBI**					
Diffuse brain injury	n (%)	27,930 (54.0%)	9,091 (53.8%)	18,839 (54.1%)	0.36
Focal brain injury	n (%)	20,008 (38.7%)	6,533 (38.7%)	13,475 (38.7%)	
Uncategorized	n (%)	3,788 ( 7.3%)	1,277 ( 7.6%)	2,511 ( 7.2%)	
**Injury site**	median (IQR)	1 (1–2)	1 (1–2)	1 (1–2)	< 0.001
1	n (%)	31,299 (60.5%)	10,962 (64.9%)	20,337 (58.4%)	< 0.001
2	n (%)	12,863 (24.9%)	3,926 (23.2%)	8,937 (25.7%)	
≥ 3	n (%)	7,564 (14.6%)	2,013 (11.9%)	5,551 (15.9%)	
**Max head AIS**	median (IQR)	4 (3–4)	4 (3–4)	4 (3–4)	< 0.001
3	n (%)	17,355 (33.6%)	5,864 (34.7%)	11,491 (33.0%)	< 0.001
4	n (%)	22,235 (43.0%)	7,346 (43.5%)	14,889 (42.8%)	
5	n (%)	12,136 (23.5%)	3,691 (21.8%)	8,445 (24.2%)	
**Operation for elevated ICP**	n (%)	9,278 (17.9%)	2,872 (17.0%)	6,406 (18.4%)	< 0.001
**Complication**					
None	n (%)	44,035 (85.1%)	14,662 (86.8%)	29,373 (84.3%)	< 0.001
Neurological	n (%)	3,095 ( 6.0%)	857 ( 5.1%)	2,238 ( 6.4%)	
Non-neurological	n (%)	4,596 ( 8.9%)	1,382 ( 8.2%)	3,214 ( 9.2%)	

*TBI* traumatic brain injury, *GCS* Glasgow Coma Scale, *BP* blood pressure, *AIS* abbreviated injury scale, *ICP* intracranial pressure, *IQR* interquartile range, *max* maximum

Blunt trauma was the most common type of injury among all patients. Falls on the ground level (35.1% [526/16901] and 29.9% [10397/34825] in females and males, respectively) were the most frequent cause of trauma, and the most common type of TBI was diffuse brain injury (53.8% [9091/16901] and 54.1% [18839/34825] in females and males, respectively). The proportion of severe cases (based on GCS scores and AIS scores on admission to the emergency department) was significantly lower in females compared to males (20.6% [3473/16901] versus 22.9% [7992/34825] for GCS, 21.8% [3691/16901] versus 24.2% [8445/34825] for AIS). Compared to males, a lower proportion of females were intoxicated with alcohol at the time of the trauma (5.8% [979/16901] versus 20.0% [6978/34825]; p < 0.001). A greater proportion of males compared to females had multiple sites of intracranial injury (23.2% [3926/34825] versus 25.7% [8937/16901] for two injury sites, and 11.9% [2013/34825] versus 15.9% [5551/16901] for three or more injury sites) and elevated intracranial pressure (ICP) requiring operative treatment (17.0% [2872/34825] versus 18.4% [6406/16901]).

**Fig. 2:**
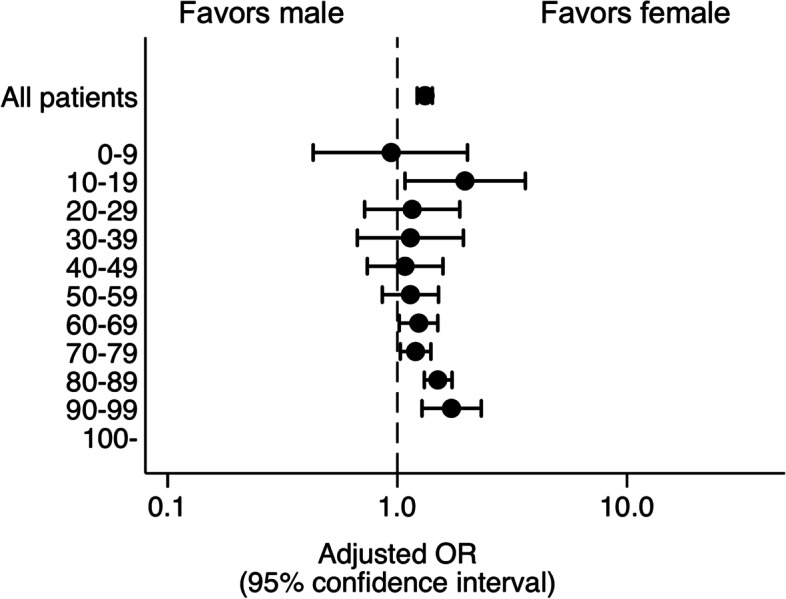
Mortality risk in males and females with traumatic brain injury within 10-year age strata. Adjusted OR for death at hospital discharge is shown. OR, odds ratio

The risk of mortality (adjusted OR, 1.32 [95% CI, 1.22–1.42] was higher for males (12.76% [4445/34825]) than females (12.01% [2030/16901]) across all ages, even after adjusting for confounders including age, injury severity, and medications (Fig. 2, Additional file 2). Sex differences in mortality risk in each 10-year age stratum are also shown in Fig. 2 and Additional file 2. A significant disparity was observed in the following age groups: 10–19 years (adjusted OR, 1.97 [95% CI, 1.08–3.61]), 60–69 years (adjusted OR, 1.24 [95% CI, 1.02–1.50]), 70–79 years (adjusted OR, 1.20 [95% CI, 1.03–1.40]), 80–89 years (adjusted OR, 1.50 [95% CI, 1.31–1.73]), and 90–99 years (adjusted OR, 1.72 [95% CI, 1.28–2.32]). Among the mechanisms of injury, back seat passenger (adjusted OR = 2.65 95% CI: 1.65–4.28) and fall from a greater height (adjusted OR = 2.82 95% CI: 2.15–3.69) were especially attributed to mortality compared to motor vehicle driver (Table 2). Table 2 also shows that neurological complication showed less effect on mortality (adjusted OR = 0.40 95% CI: 0.34–0.47) while non-neurological complication showed more effect (adjusted OR = 1.66, 95% CI: 1.52–1.82).

**Table 2:** Multivariable analysis of factors associated with the primary outcomes

	Adjusted OR	95%CI
**Gender**	1.32	(1.22–1.42)
**Age (for 10-year increment)**	1.36	(1.34–1.39)
**Year of onset (for 3-year increment)**	0.82	(0.80–0.85)
**Mechanism of injury**		
Motor vehicle driver	(reference)	
Motor vehicle passenger	1.73	(0.98–3.05)
Back seat passenger	2.65	(1.65–4.28)
Motorcycle Driver	1.18	(0.89–1.56)
Motorcycle Passenger	1.52	(0.74–3.13)
Bicycle	1.85	(1.43–2.39)
Pedestrian	2.21	(1.71–2.86)
Other vehicle	1.97	(1.12–3.44)
Fall from a great height	2.82	(2.15–3.69)
Fall down the stairs	1.82	(1.41–2.34)
Fall on the ground level	1.85	(1.45–2.37)
Other blunt injury	1.89	(1.47–2.45)
Penetrating	2.90	(1.04–8.13)
**Alcohol drunk**	0.51	(0.46–0.57)
**Anticoagulant / antiplatelet**	1.51	(1.26–1.80)
**Hypotension (BP ≤ 90) on arrival**	2.70	(2.29–3.18)
**GCS score on arrival (for 1-score increment)**	0.72	(0.72–0.73)
**Type of TBI**		
Diffuse brain injury	(reference)	
Focal brain injury	0.90	(0.82–0.99)
Uncategorized	0.84	(0.74–0.96)
**Injury site (n)**		
1	(reference)	
2	1.11	(1.01–1.23)
3 ≤	1.11	(0.99–1.25)
**Max head AIS**		
3	(reference)	
4	2.39	(2.09–2.73)
5	7.50	(6.58–8.54)
**Operation for elevated ICP**	0.69	(0.64–0.74)
**Complication**		
None	(reference)	
Neurological	0.40	(0.34–0.47)
Non-neurological	1.66	(1.52–1.82)

*TBI* traumatic brain injury, *GCS* Glasgow Coma Scale, *BP* blood pressure, *AIS* abbreviated injury scale, *ICP* intracranial pressure, *IQR* interquartile range, *max* maximum, *OR* odds ratio, *CI* confidence interval

Table 3 shows that males had an increased risk of neurological complications (e.g., diabetes insipidus, hydrocephalus, cerebrospinal fluid fistula, meningitis, post traumatic stress syndrome, cognitive dysfunction) (crude OR, 1.29 [95% CI, 1.19–1.39] and non-neurological complications (crude OR, 1.14 [95% CI, 1.07–1.22]); this was particularly evident among patients 60 years of age and over (crude OR, 1.27 [95% CI, 1.14–1.41] for neurological complications; crude OR, 1.29 [95% CI, 1.19–1.39] for non-neurological complications). Males in this age group also exhibited greater risks for respiratory complications (e.g., acute respiratory distress syndrome [ARDS]) (crude OR, 1.28 [95% CI, 1.03–1.59]), digestive complications (e.g., gastrointestinal bleeding) (crude OR, 1.34 [95% CI, 1.08–1.66]), urinary complications (e.g., acute renal dysfunction) (crude OR, 2.02 [95% CI, 1.16–3.53]), and infections (e.g., acute pneumonia, other organ infections) (crude OR, 1.47 [95% CI, 1.33–1.62]) (Table 3).

**Table 3:** Comparison of TBI complications between males and females in each age group

	**Total**	**Female**	**Male**	**Crude OR**	**(95% CI)**
**All patients**	*N* = 51,726	*N* = 16,901	*N* = 34,825		
**Neurological complication**	3,095 ( 6.0%)	857 ( 5.1%)	2,238 ( 6.4%)	1.29	(1.19–1.39)
**Non-neurological complication**	4,596 ( 8.9%)	1,382 ( 8.2%)	3,214 ( 9.2%)	1.14	(1.07–1.22)
**0—19**	*N* = 5,198	*N* = 1,522	*N* = 3,676		
**Neurological complication**	306 ( 5.9%)	86 ( 5.7%)	220 ( 6.0%)	1.06	(0.82–1.37)
**Non-neurological complication**	188 ( 3.6%)	64 ( 4.2%)	124 ( 3.4%)	0.80	(0.58–1.08)
Circulatory	26 ( 0.5%)	9 ( 0.6%)	17 ( 0.5%)	0.78	(0.35–1.76)
Respiratory	35 ( 0.7%)	10 ( 0.7%)	25 ( 0.7%)	1.04	(0.50–2.16)
Digestive	21 ( 0.4%)	4 ( 0.3%)	17 ( 0.5%)	1.76	(0.59–5.25)
Urinary	4 ( 0.1%)	1 ( 0.1%)	3 ( 0.1%)	1.24	(0.13–11.95)
Coagulation	27 ( 0.5%)	12 ( 0.8%)	15 ( 0.4%)	0.52	(0.24–1.10)
Infection	96 ( 1.8%)	30 ( 2.0%)	66 ( 1.8%)	0.91	(0.59–1.41)
**20—59**	*N* = 14,534	*N* = 3,412	*N* = 11,122		
**Neurological complication**	1,140 ( 7.8%)	238 ( 7.0%)	902 ( 8.1%)	1.18	(1.01–1.37)
**Non-neurological complication**	1,033 ( 7.1%)	229 ( 6.7%)	804 ( 7.2%)	1.08	(0.93–1.26)
Circulatory	176 ( 1.2%)	42 ( 1.2%)	134 ( 1.2%)	0.98	(0.69–1.39)
Respiratory	147 ( 1.0%)	29 ( 0.8%)	118 ( 1.1%)	1.25	(0.83–1.88)
Digestive	140 ( 1.0%)	28 ( 0.8%)	112 ( 1.0%)	1.23	(0.81–1.86)
Urinary	18 ( 0.1%)	1 ( 0.0%)	17 ( 0.2%)	5.22	(0.69–39.25)
Coagulation	107 ( 0.7%)	20 ( 0.6%)	87 ( 0.8%)	1.34	(0.82–2.18)
Infection	541 ( 3.7%)	111 ( 3.3%)	430 ( 3.9%)	1.20	(0.97–1.48)
**60 ≤ **	*N* = 31,994	*N* = 11,967	*N* = 20,027		
**Neurological complication**	1,649 ( 5.2%)	533 ( 4.5%)	1,116 ( 5.6%)	1.27	(1.14–1.41)
**Non-neurological complication**	3,375 (10.5%)	1,089 ( 9.1%)	2,286 (11.4%)	1.29	(1.19–1.39)
Circulatory	826 ( 2.6%)	292 ( 2.4%)	534 ( 2.7%)	1.10	(0.95–1.27)
Respiratory	380 ( 1.2%)	121 ( 1.0%)	259 ( 1.3%)	1.28	(1.03–1.59)
Digestive	388 ( 1.2%)	120 ( 1.0%)	268 ( 1.3%)	1.34	(1.08–1.66)
Urinary	70 ( 0.2%)	16 ( 0.1%)	54 ( 0.3%)	2.02	(1.16–3.53)
Coagulation	337 ( 1.1%)	139 ( 1.2%)	198 ( 1.0%)	0.85	(0.68–1.06)
Infection	1,927 ( 6.0%)	566 ( 4.7%)	1,361 ( 6.8%)	1.47	(1.33–1.62)

*OR* odds ratio, *CI* confidence interval

Differences in the cause and type of TBI between males and females were more evident in older age groups (Additional file 3). The proportion of TBI cases attributed to injuries sustained in traffic accidents involving back seat passenger, bicycles or pedestrians was significantly greater among females compared to males, across all age groups. While falls from a great height were significantly more often cited as the cause of TBI among adult males (crude OR, 1.66 [95% CI, 1.41–1.96] for the 20–59-year-old age group; crude OR, 4.95 [95% CI, 4.21–5.85] for the ≥ 60-year-old age group), TBI due to falls at the ground level were more common in elderly females (crude OR, 0.87 [95% CI, 0.83–0.91]). Subarachnoid hemorrhage was the leading type of TBI in both males and females, across all age groups. Focal injuries, including contusion, epidural hemorrhage, intracranial hemorrhage, and diffuse axonal injury were more common in elderly males; an exception was acute subdural hematoma, which was more frequently documented in elderly females compared to elderly males (crude OR, 0.93 [95% CI, 0.89–0.97]).

## Discussion

The results of this study indicated that sex differences in TBI-associated mortality and complications were affected by patient age. While such mortality was higher in males than females, this disparity was particularly evident among younger and older age groups. Furthermore, elderly males with TBI experienced more neurological and non-neurological complications than females of the same age.

Our finding that males had a higher mortality rate after TBI compared to females of the same age is supported by prior studies utilizing large samples (> 10,000 patients in multiple trauma centers) obtained from national registries [3]. However, some studies with smaller sample sizes have reported that females have worse clinical outcomes than males after TBI [15–17]. Gupte et al. suggested that these contrasting results could be attributed to TBI heterogeneity [3], as well as differences in TBI severity and patient age, race, and physical condition. In addition, the majority of studies which have investigated sex differences in TBI-associated mortality have included cases with injuries to other body regions [6, 18–20]. In order to remove this potentially confounding effect, we only included patients with isolated TBI. Furthermore, our study was restricted to patients in Japan; this reduced the confounding effects of race and injury type (the majority of TBI cases in Japan are attributed to blunt injuries).

Our study showed sex and age differences in TBI characteristics. TBI is typically classified according to clinical severity, with severe injuries usually categorized on the basis of a total GCS score of 8 or less, or anatomical severity based on the AIS score (3 = serious, 4 = severe, 5 = critical) [3, 6, 8]. In the present study, injury severity (in terms of both anatomical and physiological scales) was higher in males than females. This may be attributed to the greater prevalence of high-impact trauma and alcohol intoxication among males. Previous studies have reported that falls are the most common cause of TBI among older adults [21, 22]. Nevertheless, we found that TBI due to falls at the ground level were more common in elderly females compared to elderly males, while falls from greater heights were more frequently documented among elderly males (Additional file 2). Furthermore, fall from a great height was the most significant risk factor for mortality among blunt trauma (Table 2). Although we found that alcohol intake was associated with the lower mortality (adjusted OR = 0.51) in this study, the definitive mechanism was unclear. Alcohol contributes substantially to the morbidity of trauma patients, regardless of the type of injury suffered [23]. The results of this study suggest that males are more prone to severe injuries due to high-impact trauma; this may be due in large part to the effects of age and alcohol intoxication on defensive ability and alertness.

Injury type also affects mortality. Brain contusions and intracerebral, subdural, and fast-growing non-evacuated epidural hematomas pose a high risk of increased ICP and subsequent severe disability or mortality. In contrast, the risk of an increase in ICP is low for axonal injury, traumatic subarachnoid hemorrhage, and petechial bleeding [24, 25]. Subdural hematomas are typically associated with more severe types of TBI and are more frequently diagnosed in female patients [26]. Indeed, it has been reported that females are significantly more likely to experience brain swelling and intracranial hypertension than male patients with a comparable injury severity [27]. However, as the proportion of cases with multiple intracranial injury sites or elevated ICP requiring operative treatment was lower among females, we presume that the overall incidence of raised ICP was also lower compared to males.

TBI in older patients typically results from low-energy impacts such as ground-level falls, resulting in a lower proportion of subdural and epidural hematomas, as well as contusions, compared to younger patients [26]. The link between a lower incidence of raised ICP and high initial GCS scores may be explained by cerebral atrophy and an increased cerebrospinal fluid space, which may buffer new pathological intracranial masses [26]. While the JTDB did not document the deterioration of clinical symptoms, such as decreases in GCS scores by two or more points; this suggested that mortality was affected, even if the initial GCS scores on hospital admission were mild, by these factors that were not adjusted for in the present study [28]. However, as the distribution of symptom deterioration is age-dependent [28], age can significantly impact clinical outcomes by interacting with other confounding factors.

Morbidity and mortality in patients with TBI are affected by both intracranial and extracranial complications [29, 30]. Our study reveals that older males have more neurological and non-neurological complications than females of the same age. The association of non-neurological complication with higher mortality suggested that efforts to mitigate post-trauma complications might be an important target for intervention. Indeed, these complications impact in-hospital mortality, especially in elderly patients [31]. TBI triggers a complex cascade of molecular and cellular events associated with a systemic inflammatory reaction and secondary extracranial organ damage [29–31]. Circulating pro-inflammatory cytokines and chemokines following TBI are potentially responsible for ARDS and acute kidney injury [32, 33]. Patients with severe brain injury often require a deep level of sedation and the administration of neuromuscular blocking agents to control ICP [34]. The greater incidence of acute pneumonia may be partially explained as a side effect of this treatment strategy. It has been reported that acute lung injury and ARDS develop in 20–25% of patients with isolated brain injuries, and are associated with a three-fold increased risk of mortality [35]. Furthermore, it has been suggested that ARDS is an independent predictor of mortality [36]. Mortality due to ARDS in intensive care units has decreased with recent advancements in medical technology [36]; this is also likely to reduce mortality due to TBI-associated neurological complications. Nevertheless, males tend to have more complications after TBI, as well as poorer prognoses and clinical outcomes.

The causes of sex differences in mortality following TBI remain to be determined. Preclinical studies of TBI have shown inconsistent results with regard to the neuroprotective effects of sex hormones; these include the reduction of ICP, and the improvement of cerebral perfusion pressure and neurological scores [37]. It has been reported that pubescent females have lower mortality rates after isolated moderate-to-severe TBI, compared to prepubescent females [38, 39]. Taking into account published data on the mean age of menarche (12.2 + 0.9 years) and menopause (49.5 + 3.5 years) among Japanese females [40], we did not observe a lower TBI-associated mortality among females of reproductive age in the present study. This is inconsistent with a prior study that reported a better prognosis in premenopausal females due to increased levels of sex hormones [15]. On the other hand, another large multicenter study found that mortality in the > 50-year-old age group was lower in women compared to men [19]. Nevertheless, the results of clinical studies have shown that the neuroprotective mechanisms of estrogen are still controversial [41, 42]. The relative contribution of sex hormones (compared to the range of other factors identified in the present study) to sex differences in morbidity and mortality after TBI remains to be determined by future studies.

Males and females exhibit differences in terms of their developmental environment, as well as their neurodevelopment and sociological attributes; it must be acknowledged that these factors may contribute to differences in TBI incidence [43]. Our study demonstrated poorer outcomes in male TBI patients even after adjusting for various confounding factors, which may possibly be related to the intrinsic attributes of the male sex, with a greater risk of death from TBI; alternatively, these results may reflect an increased vulnerability to TBI [43]. Age was found to be an important modifier of sex differences in TBI outcomes. A better understanding of sex differences in TBI may facilitate the development of optimal treatments aimed at improving patient outcomes following TBI. The logical next step in this field of research would be to investigate sex differences in TBI cases associated with polytrauma.

### Limitations

This study has several limitations. First, the use of a retrospective study design and data from a trauma registry prevented a more detailed examination of the patient population. For example, while the type of in-hospital treatment provided (e.g., hypothermia) may have affected survival after TBI, this was not documented in the JTDB. It is reported that the use of helmet was associated with greatly reduced risk of head injury in bicycle collisions with motor vehicles, and the more severe the injury considered, the greater the reduction [44]. Therefore, wearing a helmet or a seat belt is one of important factors, although JTDB did not obtain this information. Furthermore, hypoxemia in the field or in the ED and loss of pupillary reactivity, which are reported as the most important predictors of outcome in TBI in the IMPACT study [45], were unavailable in the JTDB. Moreover, we only assessed in-hospital outcomes, and did not evaluate post-trauma quality of life. Additionally, the data included in this study primarily involved cases of blunt trauma treated in trauma centers in Japan; therefore, the results cannot be generalized to other countries which have a greater proportion of penetrating injuries and non-trauma centers. Third, unmeasured confounding factors may have influenced the association between TBI and the study outcomes. For example, patient smoking habits and the deterioration of clinical symptoms may have affected the incidence of non-neurological complications and mortality, respectively. Lastly, as with all epidemiologic studies, ascertainment bias may have affected the integrity and validity of the data; nevertheless, this was minimized with the use of uniform data collection methods based on the JTDB registration system. Furthermore, the risk of potential bias was also reduced by utilizing the largest trauma database in Japan, which provided a greater statistical power for the detection of sex differences in TBI-associated mortality.

## Conclusions

The results of this study suggested that males had a higher mortality rate than females after TBI. This disparity was especially evident among teenagers and those older than 60 years of age. Furthermore, elderly males experienced more post-TBI complications than females of the same age. The elucidation of sex differences in TBI morbidity and mortality may facilitate the development of optimal treatments aimed at improving patient outcomes.

## Supplementary Information

**Additional file 1.** Histogram of isolated traumatic brain injury patients. Sex disparity was reversed among patients 90 years of age and over

**Additional file 2.** Mortality risk in males and females with traumatic brain injury. The risk of mortality was higher for males than females across all ages, even after adjusting for confounders including age, injury severity, and medications.

**Additional file 3.** Comparison of causes and types of traumatic brain injury between males and females in each age group. Differences in the cause and type of traumatic brain injury between males and females were more evident in older age groups

## Data Availability

The data that support the findings of this study are available from the JTDB, but restrictions apply to the availability of these data, which were used under license for the current study. Therefore, the data are not publicly available. However, the data are available from the authors upon reasonable request and with permission of the JTDB.

## Abbreviations

AIS: Abbreviated Injury Scale
CI: Confidence interval
GCS: Glasgow Coma Scale
ICP: Intracranial pressure
JTDB: Japanese Trauma Data Bank
OR: Odds ratio
TBI: Traumatic brain injury
