# Supplementary material for: Sex and age differences in isolated traumatic brain injury: a retrospective observational study
Source: BMC Neurol. 2021 Jul 5;21:261. doi: 10.1186/s12883-021-02305-6 (PMC8256599; doi:10.1186/s12883-021-02305-6)
Supplement: Supplementary file 1 — Additional file 1. Histogram of isolated traumatic brain injury patients. Sex disparity was reversed among patients 90 years of age and over [file 12883_2021_2305_MOESM1_ESM.docx]

**Sex and age differences in isolated traumatic brain injury: A retrospective observational study**

Sanae Hosomi,^1,2^ Tetsuhisa Kitamura,^2^ Tomotaka Sobue,^2^ Hiroshi Ogura,^1^ Takeshi Shimazu^1^

^1^Department of Traumatology and Acute Critical Medicine, Osaka University Graduate School of Medicine, 215, Yamada-oka, Suita, Japan

^2^Division of Environmental Medicine and Population Sciences, Department of Social and Environmental Medicine, Osaka University Graduate School of Medicine, 215, Yamada-oka, Suita, Japan

**Corresponding author:** Sanae Hosomi

s-hosomi@hp-emerg.med.osaka-u.ac.jp

Department of Traumatology and Acute Critical Medicine,

Osaka University Graduate School of Medicine, 215, Yamada-oka, Suita, Japan

Additional file 1. Histogram of isolated traumatic brain injury patients.
