# Supplementary material for: Sex and age differences in isolated traumatic brain injury: a retrospective observational study
Source: BMC Neurol. 2021 Jul 5;21:261. doi: 10.1186/s12883-021-02305-6 (PMC8256599; doi:10.1186/s12883-021-02305-6)
Supplement: Supplementary file 2 — Additional file 2. Mortality risk in males and females with traumatic brain injury. The risk of mortality was higher for males than females across all ages, even after adjusting for confounders including age, injury severity, and medications. [file 12883_2021_2305_MOESM2_ESM.docx]

**Sex and age differences in isolated traumatic brain injury: A retrospective observational study**

Sanae Hosomi,^1,2^ Tetsuhisa Kitamura,^2^ Tomotaka Sobue,^2^ Hiroshi Ogura,^1^ Takeshi Shimazu^1^

^1^Department of Traumatology and Acute Critical Medicine, Osaka University Graduate School of Medicine, 215, Yamada-oka, Suita, Japan

^2^Division of Environmental Medicine and Population Sciences, Department of Social and Environmental Medicine, Osaka University Graduate School of Medicine, 215, Yamada-oka, Suita, Japan

**Corresponding author:** Sanae Hosomi

s-hosomi@hp-emerg.med.osaka-u.ac.jp

Department of Traumatology and Acute Critical Medicine,

Osaka University Graduate School of Medicine, 215, Yamada-oka, Suita, Japan

Additional file 2. Mortality risk in males and females with traumatic brain injury

| **Death at hospital discharge** | **Total** | **Female** | **Male** | **Crude OR** | **(95% CI)** | **Adjusted OR** | **(95% CI)** |
| --- | --- | --- | --- | --- | --- | --- | --- |
| All patients | 6475/51726 (12.52%) | 2030/16901 (12.01%) | 4445/34825(12.76%) | 1.07 | (1.01-1.13) | 1.32 | (1.22-1.42) |
|  |  |  |  |  |  |  |  |
| 0- | 60/2075(2.89%) | 19/672 (2.83%) | 41/1403 (2.92%) | 1.03 | (0.60-1.80) | 0.94 | (0.43-2.02) |
| 10- | 114/3123(3.65%) | 21/850 (2.47%) | 93/2273 (4.09%) | 1.68 | (1.04-2.72) | 1.97 | (1.08-3.61) |
| 20- | 180/2881(6.25%) | 44/694 (6.34%) | 136/287 (6.22%) | 0.98 | (0.69-1.39) | 1.16 | (0.72-1.87) |
| 30- | 164/2788(5.88%) | 31/626 (4.95%) | 133/2162 (6.15%) | 1.26 | (0.84-1.88) | 1.14 | (0.67-1.94) |
| 40- | 314/3776(8.32%) | 67/850 (7.88%) | 247/2926 (8.44%) | 1.08 | (0.81-1.43) | 1.08 | (0.74-1.58) |
| 50- | 543/5089(10.67%) | 128/1242 (10.31%) | 415/3847 (10.79%) | 1.05 | (0.85-1.30) | 1.14 | (0.86-1.51) |
| 60- | 1191/8891(13.40%) | 322/2609 (12.34%) | 869/6282 (13.83%) | 1.14 | (0.99-1.31) | 1.24 | (1.02-1.50) |
| 70- | 1691/11306(14.96%) | 548/4047 (13.54%) | 1143/7259 (15.75%) | 1.19 | (1.07-1.33) | 1.20 | (1.03-1.40) |
| 80- | 1815/9785(18.55%) | 658/4189 (15.71%) | 1157/5596 (20.68%) | 1.40 | (1.26-1.55) | 1.50 | (1.31-1.73) |
| 90- | 396/1962(20.18%) | 187/1089 (17.17%) | 209/873 (23.94%) | 1.52 | (1.22-1.89) | 1.72 | (1.28-2.32) |
| 100- | 7/50(14.00%) | 5/33 (15.15%) | 2/17 (11.76%) | 0.75 | (0.13-4.32) | N/A | N/A |

OR, odds ratio; CI, confidence interval
