# Supplementary material for: Sex and age differences in isolated traumatic brain injury: a retrospective observational study
Source: BMC Neurol. 2021 Jul 5;21:261. doi: 10.1186/s12883-021-02305-6 (PMC8256599; doi:10.1186/s12883-021-02305-6)
Supplement: Supplementary file 3 — Additional file 3. Comparison of causes and types of traumatic brain injury between males and females in each age group. Differences in the cause and type of traumatic brain injury between males and females were more evident in older age groups [file 12883_2021_2305_MOESM3_ESM.docx]

**Sex and age differences in isolated traumatic brain injury: A retrospective observational study**

Sanae Hosomi,^1,2^ Tetsuhisa Kitamura,^2^ Tomotaka Sobue,^2^ Hiroshi Ogura,^1^ Takeshi Shimazu^1^

^1^Department of Traumatology and Acute Critical Medicine, Osaka University Graduate School of Medicine, 215, Yamada-oka, Suita, Japan

^2^Division of Environmental Medicine and Population Sciences, Department of Social and Environmental Medicine, Osaka University Graduate School of Medicine, 215, Yamada-oka, Suita, Japan

**Corresponding author:** Sanae Hosomi

s-hosomi@hp-emerg.med.osaka-u.ac.jp

Department of Traumatology and Acute Critical Medicine,

Osaka University Graduate School of Medicine, 215, Yamada-oka, Suita, Japan

Additional file 3. Comparison of causes and types of TBI between males and females in each age group

| **Cause of TBI** | **Female** | **Male** |  |  |
| --- | --- | --- | --- | --- |
| **0 - 19** | N=1,522 | N=3,676 | **Crude OR** | **(95% CI)** |
| **Traffic accident** |  |  |  |  |
| Motor vehicle driver | 18 ( 1.2%) | 61 ( 1.7%) | 1.41 | 0.83-2.39 |
| Motor vehicle passenger | 23 ( 1.5%) | 59 ( 1.6%) | 1.06 | 0.65-1.73 |
| Back seat passenger | 69 ( 4.5%) | 104 ( 2.8%) | 0.61 | 0.45-0.84 |
| Motorcycle driver | 72 ( 4.7%) | 520 (14.1%) | 3.32 | 2.57-4.28 |
| Motorcycle passenger | 38 ( 2.5%) | 60 ( 1.6%) | 0.65 | 0.43-0.98 |
| Bicycle | 497 (32.7%) | 935 (25.4%) | 0.7 | 0.62-0.80 |
| Pedestrian | 260 (17.1%) | 457 (12.4%) | 0.69 | 0.58-0.81 |
| Other vehicle | 6 ( 0.4%) | 20 ( 0.5%) | 1.38 | 0.55-3.45 |
| **Fall** |  |  |  |  |
| Fall from a great height | 147 ( 9.7%) | 308 ( 8.4%) | 0.86 | 0.70-1.05 |
| Fall down the stairs | 161 (10.6%) | 380 (10.3%) | 0.97 | 0.80-1.18 |
| Fall on the ground level | 125 ( 8.2%) | 318 ( 8.7%) | 1.06 | 0.85-1.31 |
|  |  |  |  |  |
| **20 - 59** | N=3,412 | N=11,122 |  |  |
| **Traffic accident** |  |  |  |  |
| Motor vehicle driver | 201 ( 5.9%) | 671 ( 6.0%) | 1.03 | 0.87-1.21 |
| Motor vehicle passenger | 48 ( 1.4%) | 75 ( 0.7%) | 0.48 | 0.33-0.68 |
| Back seat passenger | 58 ( 1.7%) | 72 ( 0.6%) | 0.38 | 0.27-0.53 |
| Motorcycle driver | 308 ( 9.0%) | 1,742 (15.7%) | 1.87 | 1.65-2.13 |
| Motorcycle passenger | 36 ( 1.1%) | 40 ( 0.4%) | 0.34 | 0.22-0.53 |
| Bicycle | 797 (23.4%) | 1,270 (11.4%) | 0.42 | 0.38-0.47 |
| Pedestrian | 535 (15.7%) | 983 ( 8.8%) | 0.52 | 0.47-0.58 |
| Other vehicle | 15 ( 0.4%) | 85 ( 0.8%) | 1.74 | 1.01-3.02 |
| **Fall** |  |  |  |  |
| Fall from a great height | 184 ( 5.4%) | 963 ( 8.7%) | 1.66 | 1.41-1.96 |
| Fall down the stairs | 443 (13.0%) | 1,850 (16.6%) | 1.34 | 1.20-1.50 |
| Fall on the ground level | 519 (15.2%) | 1,917 (17.2%) | 1.16 | 1.04-1.29 |
|  |  |  |  |  |
| **60 ≤** | N=11,967 | N=20,027 |  |  |
| **Traffic accident** |  |  |  |  |
| Motor vehicle driver | 230 ( 1.9%) | 660 ( 3.3%) | 1.74 | 1.49-2.02 |
| Motor vehicle passenger | 89 ( 0.7%) | 25 ( 0.1%) | 0.17 | 0.11-0.26 |
| Back seat passenger | 115 ( 1.0%) | 39 ( 0.2%) | 0.2 | 0.14-0.29 |
| Motorcycle driver | 421 ( 3.5%) | 822 ( 4.1%) | 1.17 | 1.04-1.32 |
| Motorcycle passenger | 6 ( 0.1%) | 14 ( 0.1%) | 1.39 | 0.54-3.63 |
| Bicycle | 1,390 (11.6%) | 1,773 ( 8.9%) | 0.74 | 0.69-0.80 |
| Pedestrian | 1,577 (13.2%) | 1,403 ( 7.0%) | 0.5 | 0.46-0.54 |
| Other vehicle | 29 ( 0.2%) | 84 ( 0.4%) | 1.73 | 1.14-2.65 |
| **Fall** |  |  |  |  |
| Fall from a great height | 167 ( 1.4%) | 1,312 ( 6.6%) | 4.95 | 4.21-5.83 |
| Fall down the stairs | 1,864 (15.6%) | 3,974 (19.8%) | 1.34 | 1.26-1.43 |
| Fall on the ground level | 5,291 (44.2%) | 8,191 (40.9%) | 0.87 | 0.83-0.91 |

| **Type of TBI** | **Female** | **Male** |  |  |
| --- | --- | --- | --- | --- |
| **0 - 19** | N=1,522 | N=3,676 | **Crude OR** | **(95% CI)** |
| **Focal brain injury** |  |  |  |  |
| Contusion | 479 (31.5%) | 1,150 (31.3%) | 0.99 | 0.87-1.13 |
| Acute epidural hematoma | 404 (26.5%) | 896 (24.4%) | 0.89 | 0.78-1.02 |
| Acute subdural hematoma | 434 (28.5%) | 1,072 (29.2%) | 1.03 | 0.90-1.18 |
| Intracerebral hemorrhage | 88 ( 5.8%) | 253 ( 6.9%) | 1.2 | 0.94-1.55 |
| Other focal hematoma | 5 ( 0.3%) | 18 ( 0.5%) | 1.49 | 0.55-4.03 |
| **Diffuse brain injury** |  |  |  |  |
| Diffuse axonal injury | 132 ( 8.7%) | 323 ( 8.8%) | 1.01 | 0.82-1.25 |
| Diffuse brain swelling | 30 ( 2.0%) | 72 ( 2.0%) | 0.99 | 0.65-1.53 |
| Subarachnoid hemorrhage | 572 (37.6%) | 1,245 (33.9%) | 0.85 | 0.75-0.96 |
|  |  |  |  |  |
| **20 - 59** | N=3,412 | N=11,122 |  |  |
| **Focal brain injury** |  |  |  |  |
| Contusion | 1,128 (33.1%) | 4,224 (38.0%) | 1.24 | 1.14-1.34 |
| Acute epidural hematoma | 508 (14.9%) | 2,333 (21.0%) | 1.52 | 1.37-1.68 |
| Acute subdural hematoma | 1,046 (30.7%) | 3,450 (31.0%) | 1.02 | 0.94-1.11 |
| Intracerebral hemorrhage | 180 ( 5.3%) | 795 ( 7.1%) | 1.38 | 1.17-1.63 |
| Other focal hematoma | 20 ( 0.6%) | 72 ( 0.6%) | 1.11 | 0.67-1.82 |
| **Diffuse brain injury** |  |  |  |  |
| Diffuse axonal injury | 128 ( 3.8%) | 541 ( 4.9%) | 1.31 | 1.08-1.60 |
| Diffuse brain swelling | 76 ( 2.2%) | 234 ( 2.1%) | 0.94 | 0.73-1.23 |
| Subarachnoid hemorrhage | 2,054 (60.2%) | 6,093 (54.8%) | 0.8 | 0.74-0.87 |
|  |  |  |  |  |
| **60 ≤** | N=11,967 | N=20,027 |  |  |
| **Focal brain injury** |  |  |  |  |
| Contusion | 3,201 (26.7%) | 7,219 (36.0%) | 1.54 | 1.47-1.62 |
| Acute epidural hematoma | 880 ( 7.4%) | 2,032 (10.1%) | 1.42 | 1.31-1.55 |
| Acute subdural hematoma | 5,391 (45.0%) | 8,657 (43.2%) | 0.93 | 0.89-0.97 |
| Intracerebral hemorrhage | 663 ( 5.5%) | 1,454 ( 7.3%) | 1.33 | 1.21-1.47 |
| Other focal hematoma | 92 ( 0.8%) | 170 ( 0.8%) | 1.11 | 0.86-1.43 |
| **Diffuse brain injury** |  |  |  |  |
| Diffuse axonal injury | 142 ( 1.2%) | 361 ( 1.8%) | 1.53 | 1.26-1.86 |
| Diffuse brain swelling | 180 ( 1.5%) | 299 ( 1.5%) | 0.99 | 0.82-1.20 |
| Subarachnoid hemorrhage | 6,157 (51.4%) | 10,637 (53.1%) | 1.07 | 1.02-1.12 |

TBI = traumatic brain injury
